# Prevalence of polymorphisms in *OPG*, *RANKL* and *RANK* as potential markers for Charcot arthropathy development

**DOI:** 10.1038/s41598-017-00563-4

**Published:** 2017-03-29

**Authors:** Bożena Bruhn-Olszewska, Anna Korzon-Burakowska, Grzegorz Węgrzyn, Joanna Jakóbkiewicz-Banecka

**Affiliations:** 1 Department of Molecular Biology, Wita Stwosza 59, 80-308 Gdańsk, Poland; 2 Department of Diabetology and Hypertension, Medical University of Gdańsk, Gdańsk, Poland; 3 Department of Medical Biology and Genetics, University of Gdańsk, Wita Stwosza 59, 80-308 Gdańsk, Poland; 4 Department of Bacterial Molecular Genetics, University of Gdańsk, Wita Stwosza 59, Gdańsk, 80-308 Poland

## Abstract

Charcot arthropathy is one of the most serious complications of diabetic foot syndrome that leads to amputation of the affected limb. Since there is no cure for Charcot arthropathy, early diagnosis and implementation preventive care are the best available treatment. However, diagnosis is hindered by obscure clinical picture of the disease and lack of molecular markers for its early detection. Results of recent research suggest that OPG-RANKL-RANK axis regulating bone metabolism can be associated with Charcot arthropathy and that SNPs in *OPG* gene are associated with the disease. Here we report the results of comprehensive analysis of ten SNPs in *OPG*, *RANKL* and *RANK* genes in 260 subjects divided into diabetes, neuropathy and Charcot arthropathy groups. Besides genotype analysis we performed linkage disequilibrium and hierarchical clustering to obtain information about correlation between SNPs. Our results show that *OPG* 245T/G (rs3134069) and *OPG* 1217C/T (rs3102734) polymorphisms co-occur in patients with Charcot arthropathy (r2 = 0.99). Moreover, hierarchical clustering revealed a characteristic profile of all SNPs in Charcot arthropathy and neuropathy, which is distinct from control group. Our results suggest that analysis of multiple SNPs can be used as potential marker of Charcot arthropathy and provide insight into possible molecular mechanisms of its development.

## Introduction

Diabetes is one of the most common chronic diseases occurring in the twenty-first century^1^. This disease has a major impact on the body metabolism, which results in numerous health complications accompanying diabetes and contributing to its mortality such as increased risk of heart disease and stroke^2, 3^. Moreover, health complications resulting of diabetes often develop into complex disorders like diabetic foot syndrome, which is one of the major causes of the non-traumatic lower limb amputation^4^. It is characterized by presence of neuropathy, foot ulcer and subsequent infections. In addition, neuropathy symptoms such as tingling, pain in the foot and then a partial loss of sensation, often lead to the neuropathic foot deformities, and can develop into the Charcot arthropathy (or Charcot neuroosteoarthropathy – CN)^5, 6^. It is characterized by deformation of the foot shape due to progressive inflammation of joints and soft tissue, accompanied by decreased bone density, which altogether lead to amputation of the affected limb^7^. Although CN develops as a consequence of neuropathy, presence of inflammation and abnormal bone deformation indicate that additional factors are responsible for its occurrence. Details of pathogenesis of CN are still unknown but the available data indicate that there is a strong link between OPG/RANKL/RANK axis and this disease^8, 9^.

The RANKL protein (receptor activator of nuclear factor NF-κB ligand) is produced by the osteoblastic cell line (mature osteoblasts and their precursors), chondrocytes, fibroblasts and activated T lymphocytes^10, 11^. The most important step of the osteoclastogenesis is binding of RANKL to its RANK receptor, anchored in the cell membrane of preosteoclasts. On the other hand, osteoprotegerin (OPG) is a cytokine synthesized and secreted by activated osteoblasts. This protein acts as a decoy receptor for RANKL and prevents binding of RANKL to RANK. Binding of RANKL to OPG results in the inhibition of bone resorption and stimulates bone mass building. The dynamic equilibrium between RANKL and OPG concentrations is crucial for normal bone metabolism^12^. Alternatively, the imbalance of the RANKL/OPG ratio could lead to an uncontrolled loss of bone mass^13^.

Diabetes-induced distortion of the RANKL/OPG ratio can directly affect bone formation and regeneration, i.e. processes that are likely impaired in CN^7^. Moreover, altered levels of RANKL and OPG have been linked to the development of vascular calcification, which has been proposed as one of the causes of this disease^9, 14^. A recent study indicated that the observed bone destruction in CN could be partially caused by an increase in the *RANKL* gene expression and subsequent local inflammation in the affected limb^15^. It has been also proposed that an increased bone resorption and calcification of blood vessels could lead to the development of Charcot arthropathy or intensify the development of this disease^9, 15, 16^. Therefore, increased RANKL/OPG ratio can be both, a symptom and a cause in the development of the Charcot arthropathy.

Recent studies revealed CN-specific occurrence of certain polymorphisms in *OPG*, *RANKL* and *RANK* genes^17–19^. Therefore, the aim of this study was to determine the usefulness of selected SNPs as potential genetic markers of CN, allowing to assess the risk of CN development in patients with diabetes. We have analysed ten polymorphisms in the *OPG*, *RANKL* and *RANK* genes in patients with CN, diabetic neuropathy (N) and diabetes without symptoms of neuropathy or CN (D) (blood samples were collected and analysed from 77 (CN), 77 (N) and 106 (D) patients). The study design was focused on finding difference in the specific SNP occurrence between patients with the diabetic neuropathy and Charcot arthropathy, in order to find genetic markers of the disease. In addition, hierarchical clustering analysis of all analysed polymorphisms in patients provided general overview of genetic variants associated with Charcot arthropathy. In parallel to genetic analysis, serum level of RANKL and OPG was measured to gain comprehensive view on Charcot arthropathy in relation to neuropathy and diabetes. Obtained results show that combination of the genetic analysis with assessment of the RANKL and OPG serum levels could be used as a prediction marker for the development of CN in diabetic patients.

## Results

### Analysis of selected polymorphisms in *OPG*, *RANKL* and *RANK* genes

**Figure 1:**
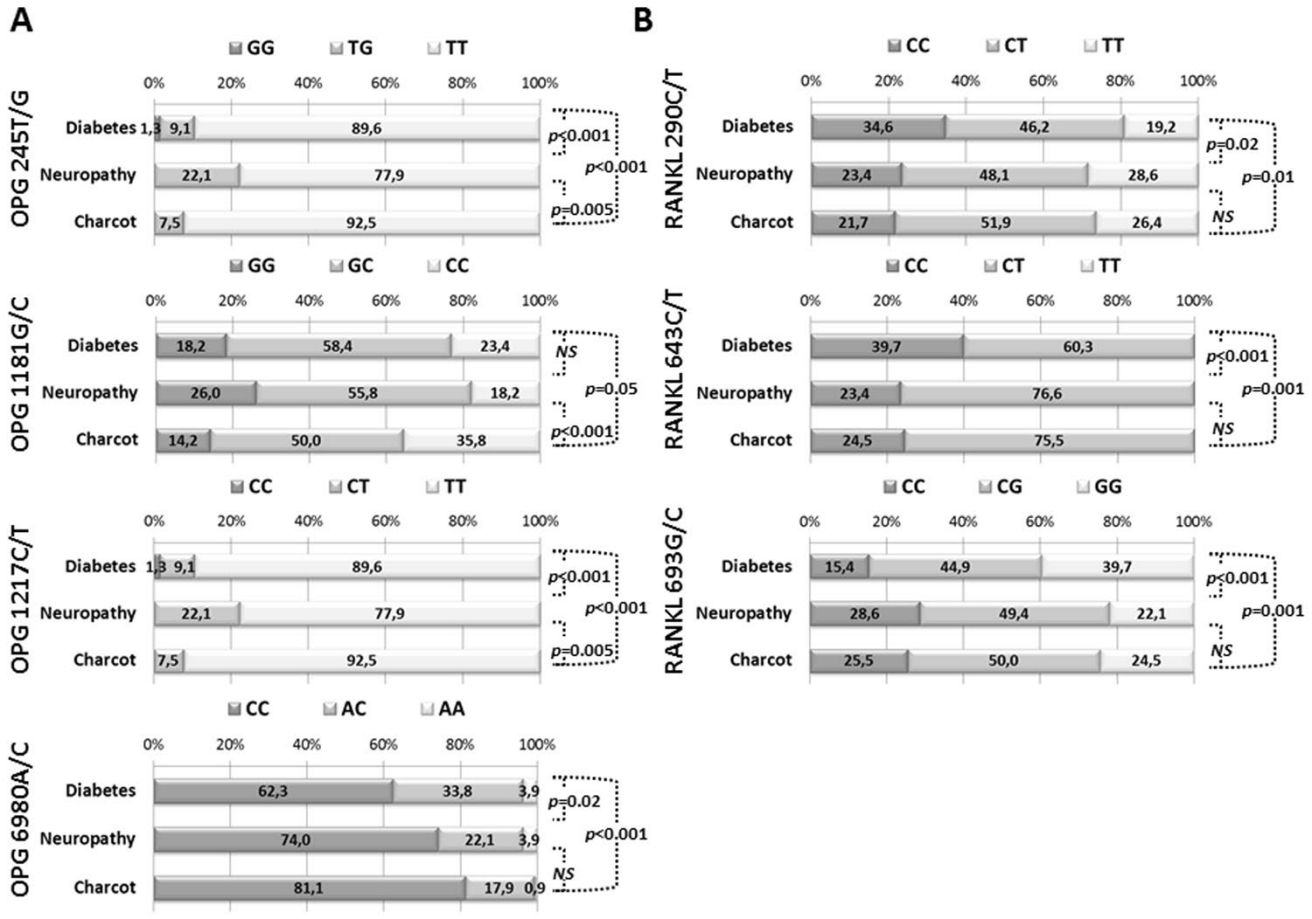
Distribution of genotypes at selected polymorphic *loci*, in the studied groups of patients. Stacked bar charts present percentage of each genotype at a given *loci* in patients with diabetes, neuropathy and Charcot arthropathy. Polymorphisms in the *OPG* gene are listed in panel A and polymorphisms in *RANKL* are listed in panel B. The calculated differences have been tested with the chi square test and *p* values for a given comparison are presented on the right side of each graph; NS stands for not significant. Only *loci* with significant difference in distribution are presented.

In this study, we analysed ten polymorphisms in three genes: *OPG* (five variants), *RANKL* (three variants) and *RANK* (two variants), in samples collected from 260 patients. Results of this analysis are summarized in Table 1. For three out of ten analysed *loci*, namely *OPG* 950T/C, *RANK* 421C/T and *RANK* 575C/T we found no significant changes in the genotype and allele frequencies across the studied groups. Out of remaining SNPs, we observed the strongest change for the C allele frequency at *OPG* 1181C/G in patients with CN. In patients with CN, the frequency of the CC genotype was higher than in patients in the N and D groups (CN-36%, N-15%, D-23%, Fig. 1A). Statistical significance of observed differences was tested by the chi-square test (p < 0.001 and p < 0.05 for CN vs. D, and CN vs. N, respectively). Similarly, we observed a bias in allele and genotype frequency towards N and CN groups for the *OPG* 6890A/C allele (Table 1 and Fig. 1A). At this *locus* the frequency of the C allele was increased in N and CN groups, what corresponds with higher occurrence of the CC genotype. In case of *OPG* 245T/G and *OPG* 1217C/T alleles the analysis revealed a higher frequency of the G and C alleles, respectively (Table 1). Interestingly, genotypes distribution at the *OPG* 245T/G and 1217C/T SNPs was identical among the phenotypic groups (Fig. 1A). The TT homozygote was the most dominant in both *loci* for all groups, whereas the GG and CC homozygotes (at *OPG* 245T/G and 1217C/T, respectively) were detected only in the diabetes group. At both *loci*, we observed that the frequency of heterozygotes is the highest in the N group.

**Table 1:** Summary of SNPs used in the study.

Gene/SNP	Common name	Chromosomal positon	Observed alleles	Observed allele frequency		
				Diabetes	Neuropathy	Charcot arthropathy
***OPG***						
**rs3134069** ^**a**^	**OPG 245T/G**	**ch**. **8 119964988**	**T**	**0**.**94**	**0**.**89**	**0**.**96**
			**G**	**0**.**06**	**0**.**11**	**0**.**04**
rs2073617	OPG 950T/C	ch. 8 119964283	T	0.51	0.55	0.59
			C	0.49	0.45	0.41
**rs2073618** ^**a**^	**OPG 1181G/C**	**ch**. **8 119964052**	**G**	**0**.**47**	**0**.**46**	**0**.**39**
			**C**	**0**.**53**	**0**.**54**	**0**.**61**
**rs3102734** ^**a**^	**OPG 1217C/T**	**ch**. **8 119964016**	**C**	**0**.**06**	**0**.**11**	**0**.**04**
			**T**	**0**.**94**	**0**.**89**	**0**.**96**
**rs7844539** ^**a**^	**OPG 6890A/C**	**ch**. **8 119938725**	**A**	**0**.**21**	**0**.**15**	**0**.**10**
			**C**	**0**.**79**	**0**.**85**	**0**.**9**
***RANKL***						
**rs9525641** ^**a**^	**RANKL 290C/T**	**ch**. **13 43148024**	**C**	**0**.**58**	**0**.**47**	**0**.**48**
			**T**	**0**.**42**	**0**.**53**	**0**.**52**
**rs9533156** ^**a**^	**RANKL 643C/T**	**ch**. **13 43147671**	**C**	**0**.**70**	**0**.**62**	**0**.**62**
			**T**	**0**.**30**	**0**.**38**	**0**.**38**
**rs9533155** ^**a**^	**RANKL 693G/C**	**ch**. **13 43147621**	**G**	**0**.**62**	**0**.**47**	**0**.**50**
			**C**	**0**.**38**	**0**.**53**	**0**.**50**
***RANK***						
rs1805034	RANK 421C/T	ch. 28 60021761	C	0.90	0.84	0.89
			T	0.10	0.16	0.11
rs35211496	RANK 575C/T	ch. 28 60027241	C	0.47	0.45	0.51
			T	0.53	0.55	0.49

^a^Source NCBI dbSNP Population diversity (http://www.ncbi.nlm.nih.gov/SNP/).

*Loci* showing a statistically significant allele frequency are in bold and with grey background.

“Allele frequency” denotes values obtained in this study from samples of the Diabetes, Neuropathy and Charcot arthropathy patient groups.

The analysis of *RANKL* 290C/T, 643C/T and 693G/C polymorphisms also revealed differences in the allele and genotype frequencies among the studied groups (Table 1 and Fig. 1B). The T allele in *RANKL* 290C/T and 643C/T, as well as the C allele in 693G/C, were more frequent in the N and CN groups. It correlates with the increased frequency of the corresponding homozygotes in those groups (TT for 290C/T and 643C/T, and CC for 693G/C). Statistical analysis of the genotype frequencies indicated that N and CN groups differ significantly from the D group (Fig. 1B).

### Linkage disequilibrium analysis

**Figure 2:**
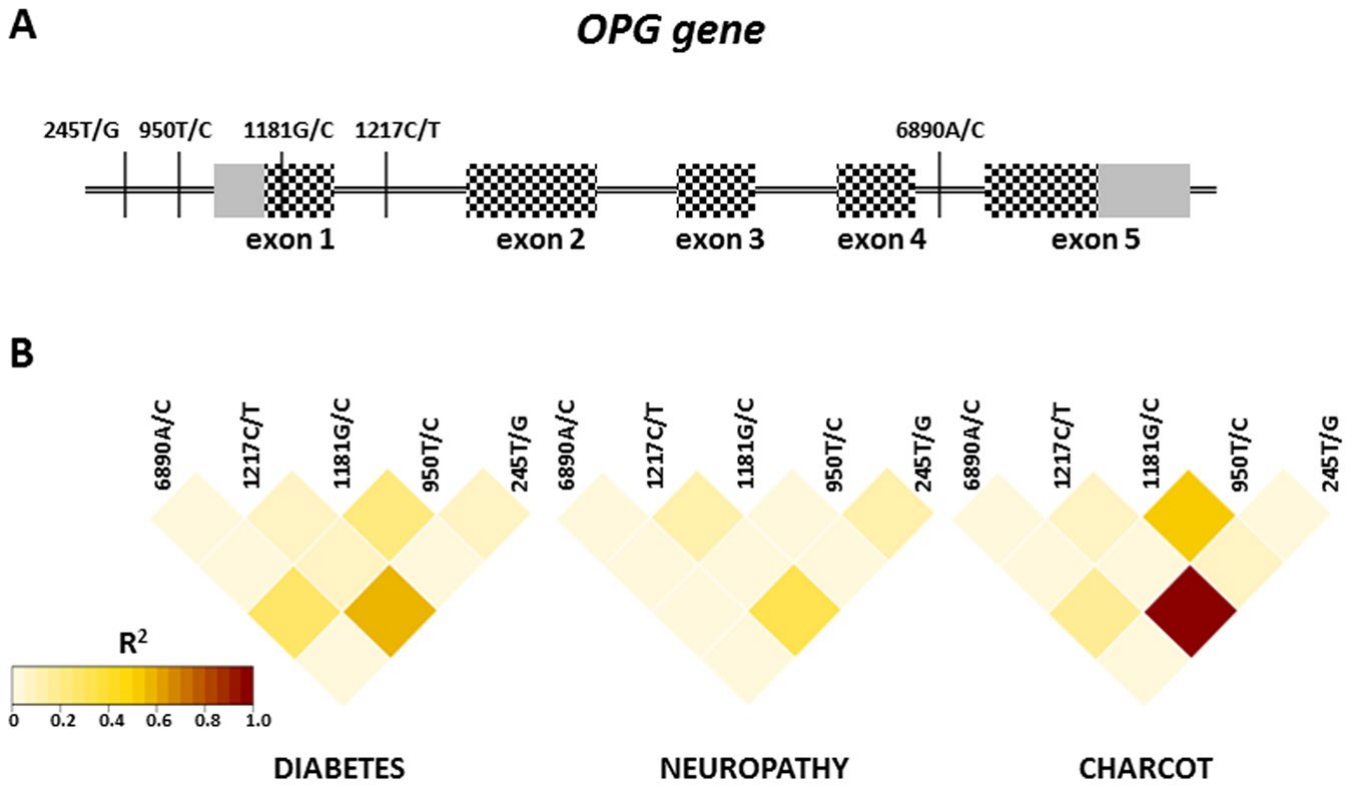
Linkage disequilibrium analysis for selected polymorphisms in the *OPG* gene. (**A**) Schematic representation of the *OPG* gene with position of studied polymorphisms. Exons are marked as boxes, with coding sequence marked as chessboard. (**B**) Linkage disequilibrium heat-maps are based on the r^2^ value, calculated for each pair. Color intensity corresponds to higher r^2^ values, according to the legend in the bottom left corner.

**Figure 3:**
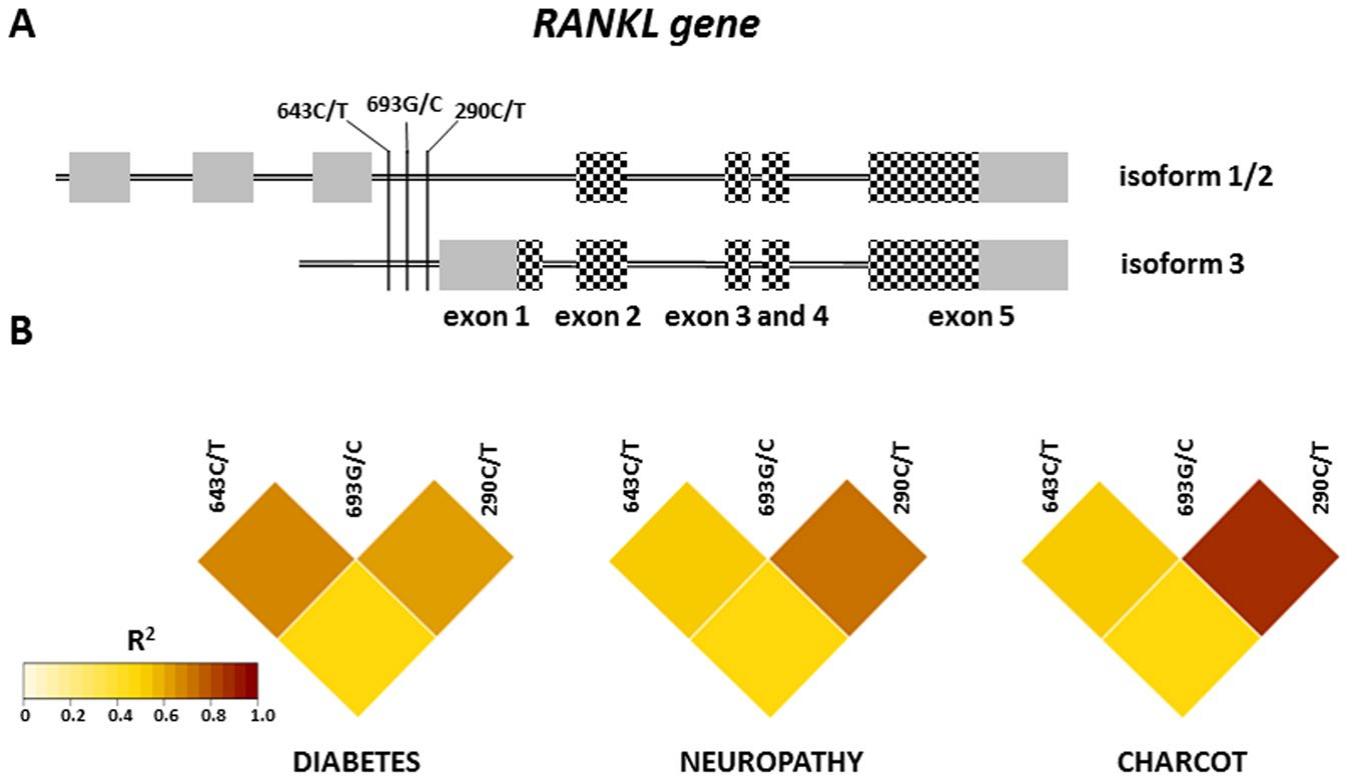
Linkage disequilibrium analysis for selected polymorphisms in the *RANKL* gene. (**A**) Schematic representation of the *RANKL* gene with position of studied polymorphisms. Exons are marked as boxes, with coding sequence marked as chessboard. (**B**) Linkage disequilibrium heat-maps basing on the r^2^ value, calculated for each pair. Color intensity corresponds to higher r^2^ values according to the legend in the bottom left corner.

The analysis of SNPs revealed that some alleles and genotypes have similar patterns of distribution in studied groups. In order to determine if these patterns are specific for a given condition, we calculated linkage disequilibrium (LD) for the analysed SNPs in the *OPG* and *RANKL* genes (Figs 2 and 3, respectively). For *OPG* gene polymorphisms we observed the highest LD between *OPG* 245T/G and *OPG* 1217C/T for the CN group (r^2^ = 0.99) and lower values for D and N groups (r^2^ = 0.58 and r^2^ = 0.34 respectively). A weaker association was observed between *OPG* 1181G/C and 950T/C in CN group (r^2^ = 0.51), although it was still higher than in D and N groups (r^2^ = 0.24 and r^2^ = 0.04 respectively).

The same analysis was performed for the *RANKL* gene polymorphisms and has revealed the highest LD to be between *RANKL* 693G/C and 290C/T polymorphisms in the CN group (r^2^ = 0.89, Fig. 3) but in other groups the their association was also high (r^2^ = 0.60 and r^2^ = 0.72 in D and N group respectively). In case of *RANKL* 693G/C and 643C/T we observed the lowest association in CN group (r^2^ = 0.52) while it was the highest in D group (r^2^ = 0.69). The LD analysis has been also performed between *OPG* and *RANKL* polymorphisms but did not show disequilibrium at any of the studied groups.

### Hierarchical clustering of SNPs in the *OPG*, *RANKL* and *RANK* genes

**Figure 4:**
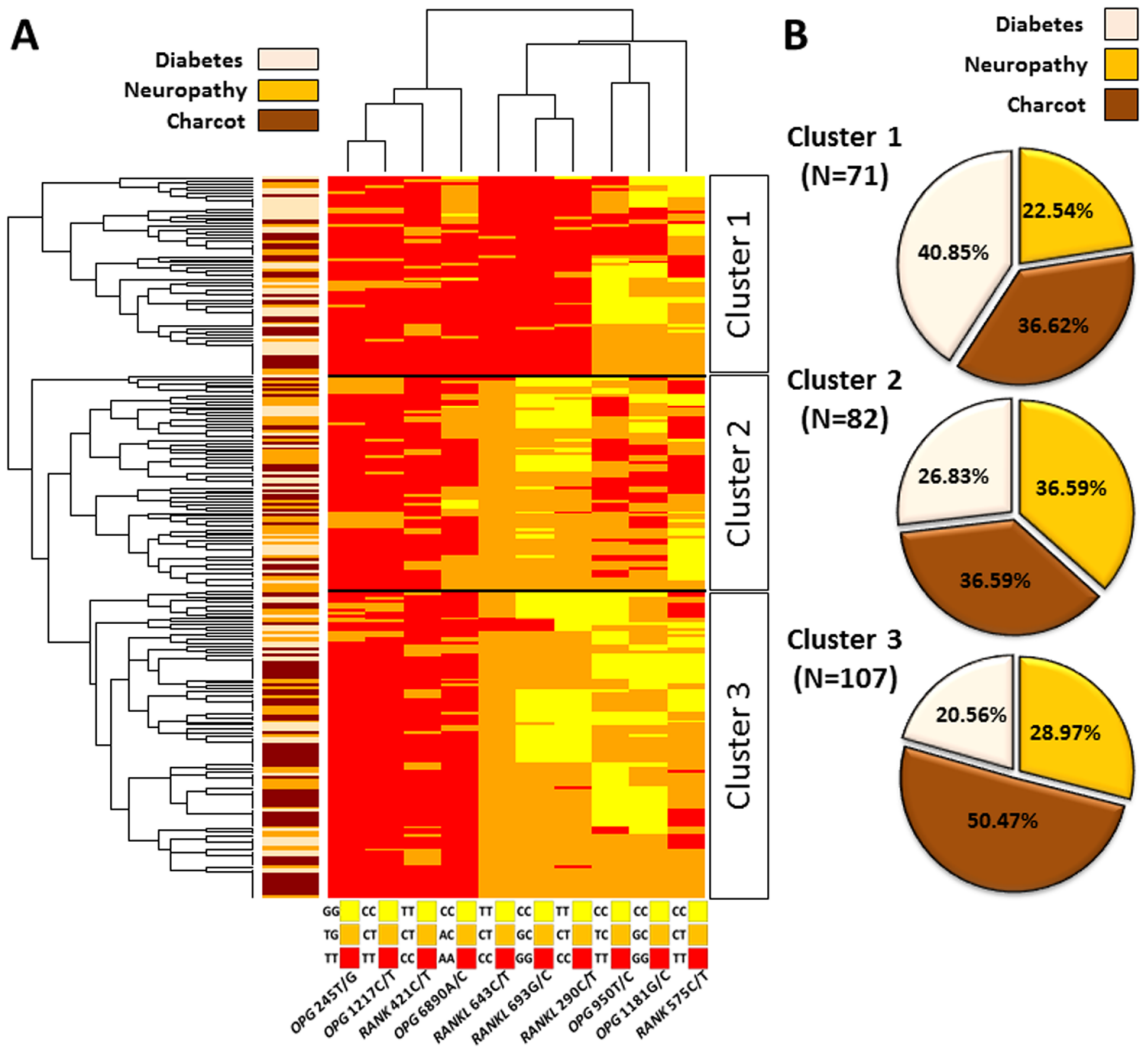
Hierarchical clustering of SNPs in *OPG*, *RANKL* and *RANK* genes. (**A**) Distribution of SNPs in selected genes is shown as a heat map where colors correspond to homozygote or heterozygote, accordingly to the legend for each SNP (below the heat map). Row dendrogram shows patient SNP profile clusters and column dendrogram shows distribution pattern of selected SNPs across all patients. Side bar color corresponds to a group according to the legend above the bar. 260 patients have been divided into three clusters according to the similarities in their SNPs’ profiles. (**B**) Percentage of patients diagnosed with diabetes, neuropathy or Charcot arthropathy in the corresponding clusters. The legend is shown above the pie charts. Numbers in brackets below the percentage show the number of patients from each cluster.

Human diseases rarely depend on single genetic variants and are likely a cumulative effect of multiple changes in different genes. Therefore analysis of the occurrence of multiple SNPs in patients is more suitable for finding genetic basis of a disease. In order to obtain a general overview of genotype distribution in the Charcot arthropathy, we have performed hierarchical clustering (HC) analysis for all analysed polymorphisms in the studied group of patients (Fig. 4A). The genotype data has been transformed to generate a matrix where each row represents a patient and each column represents a SNP. Both, rows and columns, have been sorted on the basis of the genotypes occurrence similarity, resulting in clusters depicted as dendrograms. The major advantage of HC is that it shows similarities between patients (rows) and SNPs (columns). Consistently with the LD analysis (Fig. 2B), *OPG* 245T/G and *OPG* 1217C/T have nearly identical pattern of distribution. However, *RANK* 421C/T and *OPG* 6890A/C also cluster together with *OPG* 245T/G and 1217C/T, which was not detected by pairwise comparison using only LD. All three *RANKL* SNPs analysed here, have been grouped together and patterns of genotypes at these positions fit well with the three distinguished clusters. Analysis of the patient study group designation revealed that the three clusters are comprised in different proportions of D, N and CN patients (Fig. 4B). The first cluster is composed mostly of the D patients (40.85%), whereas in the second and third cluster, the D patients comprise 26.83% and 20.56%, respectively. The same proportion of N and CN patients (36.59%) characterizes the second cluster. The highest number of patients with CN has been observed in the third cluster (50.47%) (Fig. 4B).

### Cytokine concentration in the blood serum

**Figure 5:**
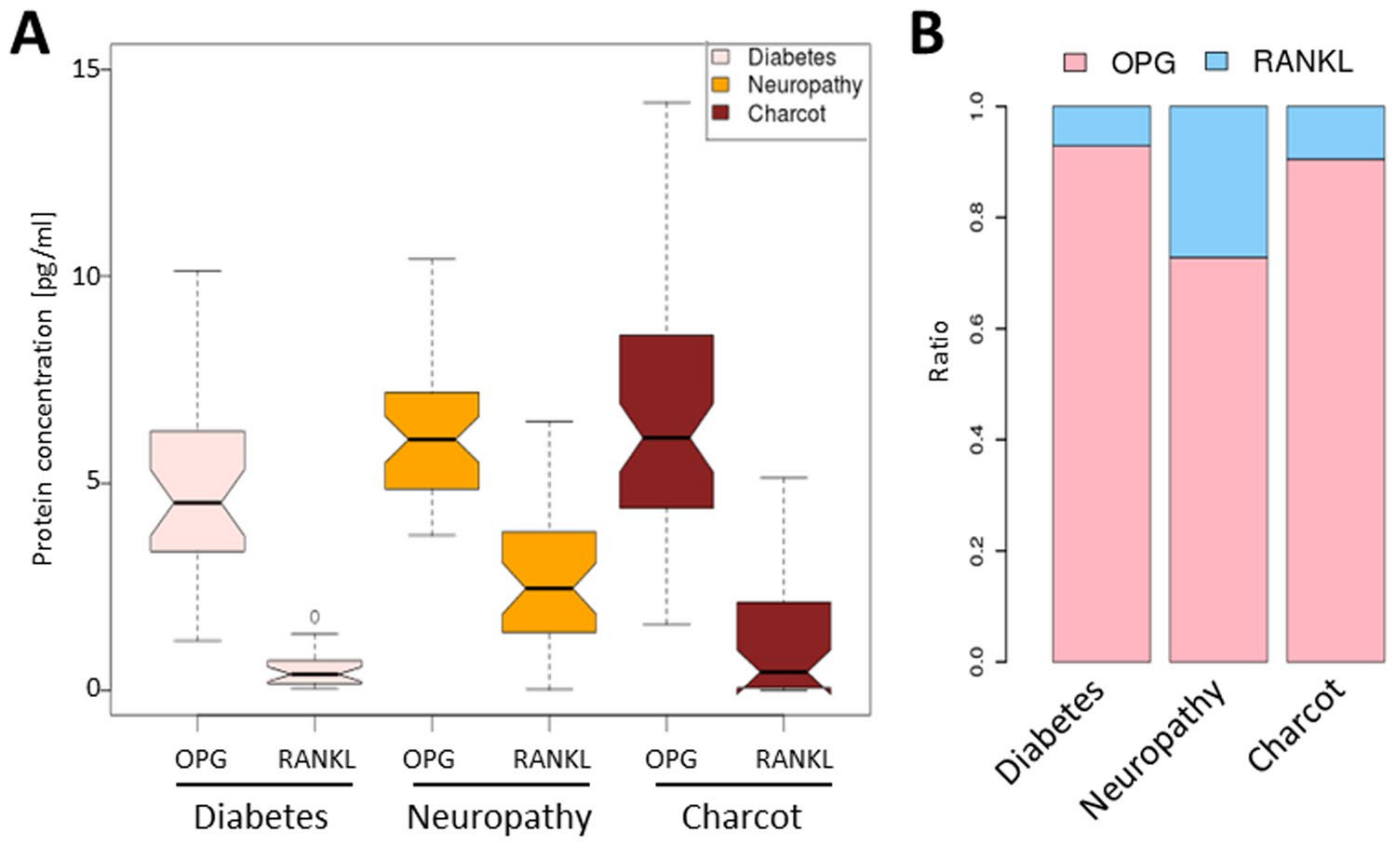
RANKL and OPG protein concentration in the blood serum. Results have been obtained with ELISA (for details see Materials and methods). (**A**) OPG and RANKL proteins levels in blood serum of patients from each group. Groups have been colored according to the legend in the top right corner. (**B**) Stacked bar plots showing proportion of RANKL (blue) and OPG (rose) in each group of patients. Bars represent percentage of summarized OPG and RANKL protein concentration for each protein.

It has been reported that in the CN patients an increased ratio of RANKL to OPG in the blood serum is observed in respect to healthy subjects. Therefore, we have determined levels of these cytokines in the blood serum of the three examined groups of patients, using commercial ELISA assays. We observed increased levels of RANKL in CN and N groups comparing to D group (1.01 ± 1.45 pmol/l, 2.66 ± 1.74 pmol/l and 0.5 ± 0.43 pmol/l respectively, Fig. 5A). Surprisingly, the highest concentration of the protein was in N group, what has not been observed before. Observed differences in OPG concentration were statistically significant as determined by T-test (p < 0.01 for CN vs. N and CN vs. D, p < 0.001 for N vs. D).

The OPG levels were higher in patients in the CN and N groups, when comparing to the D group (7.36 ± 4.1 pmol/l in CN, 6.29 ± 1.68 pmol/l in N and 4.77 ± 2.38 pmol/l in D, Fig. 5A). These differences were statistically significant as indicated by T-test (p < 0.01 for N vs. D and p < 0.001 for CN vs. D), while there was no difference between CN and D groups. The proportion of RANKL to OPG was the highest in the N group (0.42) and was approximately 4-fold higher than in D group (0.10), whereas in the CN group (0.14) the ratio of RANKL to OPG was approximately 1.5-fold higher than in the D control (Fig. 5B).

## Discussion

The pathogenesis of Charcot arthropathy is still unknown but research on the OPG/RANKL/RANK cytokines axis has highlighted this system’s role in the bone-associated diseases. In this work, we have performed a comprehensive genetic analysis of ten polymorphic *loci* in the 260 patients, divided into three groups (106 CN, 77 N and 77 D). To our knowledge, this is the largest group of patients studied in the context of Charcot arthropathy and neuropathy in diabetes. In parallel to classical genotyping, we present here hierarchical clustering as a useful analysis in the studies of SNP association with human disease. Our results suggest association of the *RANKL* gene polymorphisms with N (neuropathy) and CN (Charcot arthropathy). Moreover, we suggest that increased ratio of RANKL/OPG in the blood serum is specific for neuropathy and can be a factor leading to the development of Charcot arthropathy.

To date, studies addressing a possible link between genotype and Charcot arthropathy have been limited to the analysis of the *OPG* gene polymorphisms^17, 18^. In the study presented here, we have analysed additional SNPs that have been associated with altered bone metabolism in postmenopausal women^20, 21^. In total, out of ten selected SNPs three did not show any association with either of the studied conditions. Our results indicate that *OPG* 950T/C, and *RANK* 421C/T and 575C/T, which have been previously analysed in the context of Paget’s disease and osteoporosis, are not associated with CN^21, 22^. For the remaining seven SNPs, four in the *OPG* gene (245T/G, 1181G/C, 1217C/T and 6890A/C) and three in the *RANKL* gene (290C/T, 643C/T and 693G/C), we have found statistically significant differences in the genotype distribution in the studied groups.

Consistently with previous reports, the strongest association with CN was observed for the *OPG* 1181G/C polymorphism^17, 18^. Moreover, although the hierarchical clustering did not show any characteristic *OPG* 1181G/C genotype pattern for a particular patient cluster, the majority of patients with the CC genotype belongs to the CN and N groups (Fig. 4A). Interestingly, *OPG* 1181G/C is located in the coding sequence of this gene and point mutations result in the codon change from lysine into asparagine (Fig. 2A). This amino acid substitution could potentially influence the protein activity, explaining its association with CN. Intriguing is also the co-occurrence of *OPG* 1181G/C and 950T/C in CN. The later polymorphism is located upstream from transcription start site and its role in the disease and in association with *OPG* 1181G/C is unknown. It is also challenging to propose a mechanism explaining observed association between *OPG* 245T/G and 1217C/T with CN and N (Fig. 2B). Both SNPs are located in the noncoding region of this gene, with *OPG* 245T/G located in the putative promoter region of the *OPG* gene and 1217C/T located in the first intron (Fig. 2A). This location, especially that of *OPG* 245T/G, would seem to suggest altered gene expression as the underlying cause of the observed phenotypes, however, since little is known about molecular mechanisms underlying *OPG* gene expression, it is unknown if SNP variants at *OPG* 245 and 1217 can influence this process. Yet, both *loci* show strong linkage disequilibrium and very similar pattern of genotype distribution in the studied patients (Figs 2B and 4A).

In contrast to previous studies^17, 18^, we aimed to find differences between CN, N and the D group, which was used as the control group. This distinction allowed for finding that *OPG* 6890A/C is associated with both, CN and N. However, as in the case of *OPG* 1217C/T, this SNP is located in the intron sequence and it is unclear how this position contributes to the development of CN and N. Furthermore, *OPG* 6890A/C was clustered together with *OPG* 1217C/T, *OPG* 245T/G and *RANK* 421C/T polymorphisms, which did not show distribution patterns (Fig. 4A, upper dendrogram).

Our results also show that *RANKL* 290C/T, 643C/T and 693G/C polymorphisms are associated with both, CN and N (Fig. 1B). All three polymorphisms show similar distribution across all patients and form a separate cluster in the HC analysis (Fig. 4A, upper dendrogram). The general distribution pattern of these polymorphisms fits well with cluster 1 and clusters 2 and 3. Cluster 1, where diabetic patients are the most abundant group (40.85%, Fig. 4B) is mostly composed of genotypes CC, GG, CC for *RANKL* 643C/T, 693G/C and 290C/T polymorphisms respectively. On the contrary, in clusters 2 and 3 where Charcot patients comprise 36.59% and 50.47% respectively, heterozygotes and homozygotes TT, CC and TT for aforementioned SNPs are the most frequent. This suggests that all studied *RANKL* polymorphisms can be associated with either CN alone or CN and N. Noteworthy, all three SNPs are located in the putative promoter region of *RANKL* what raises the possibility that sequence variation at these *loci* affects the gene expression. As revealed by LD analysis, there is an association between *RANKL* 693G/C and 290C/T co-occurrence in CN (Fig. 3B). On the contrary, the other pair of *RANKL* SNPs, 643C/T and 693G/C shows decreased association in CN in respect to D and N groups. However, it is only a hypothesis and further studies are required to determine if SNPs combination affects the expression of the *RANKL* gene. It has been proposed that bone loss observed in CN is a result of inflammation-induced *RANKL* expression^22^. It would be interesting to test whether SNPs in the promoter region enhance stimulation of *RANKL* expression by the tumour necrosis factor α (TNF-α).

Although it is unknown if SNPs can influence *RNAKL* expression, it has been reported that CN patients show increased levels of RANKL protein in the blood serum^23^. In agreement with this report, we found increased level of RANKL in patients with N and CN. However, in our analysis RANKL concentration is mostly increased in the blood serum of N group, resulting in nearly 4-fold higher RANKL/OPG ratio than in the diabetic patients (Fig. 5A and B). It is likely that the elevated level of RANKL during the neuropathy stage is the major factor responsible for the bone loss observed in the Charcot arthropathy. Interestingly, we observed that the neuropathy group in our study showed lower RANKL/OPG ratio, due to high level of OPG which can counterbalance bone loss triggered by RANKL. Currently it is unknown if this observation is of clinical significance and can be a marker of neuropathy or reflects a stage during Charcot arthorpathy development.

Based on the results reported here, it is possible that at least two molecular mechanisms underlie the development of CN. The first one involves the OPG protein, which is required for quenching serum-soluble RANKL. Changes in the protein sequence can affect its activity and result in increased bone resorption due to RANKL-induced osteoclastogenesis. A second mechanism can be directly related to the *RANKL* gene expression, which can be influenced by the DNA sequence in the proximity of the promoter. SNPs in this position could directly alter the *RANKL* gene expression, or could enhance response to the TNF-α-mediated *RANKL* expression activation. Both mechanisms are likely and are supported by observed, increased levels of RANKL in the blood serum. However, regulation of human genes expression rarely depends solely on promoter activity and involves additional levels of regulation, including among others DNA methylation and post-transcriptional regulation by miRNA, which may underlie the phenotypes observed in diabetes and its complications^24^. Recently, it has been shown that overall DNA methylation level is decreased in fibroblasts derived from diabetic foot patients^25^. As Charcot arthropathy is a consequence of diabetic foot syndrome, it is likely that its development is also related with changed methylation pattern. Although results of methylation status in fibroblasts indicate that it affects genes associated with wound healing^25^, it would be interesting to analyse methylation status in bone cells and correlate methylation patterns with gene expression analysis to obtain a comprehensive view on the epigenetic mechanism of Charcot arthropathy development. Integrating transcriptome analysis in such study should include miRNA analysis, since non-coding RNAs are often factors in human disease^26^. Currently there is no data on changes in transcriptome in Charcot arthropathy or on activity of non-coding RNAs in this particular disease. However, there are reports indicating that individual miRNAs can affect levels of *RANKL* and *RANK* mRNAs. It has been shown that upregulation of miR-18a results in decreased level of RANKL mRNA in hepatocellular carcinoma^27^. Recently, miR-503 has been shown to regulate RANK levels and down regulation of miR-503 results in bone loss due to increased osteoclastogenesis^28^. Considering the above, it is likely that miRNAs play a role in the development of Charcot arthropathy and testing this hypothesis should be one of the next steps in dissecting mechanisms of this condition.

In summary, our work provides an insight into genetic changes associated with Charcot arthropathy. Analysis of *loci* presented in this work could be potentially used to predict risk of Charcot arthropathy development in patients with diabetes, especially in multiplex analysis. We also suggest possible molecular mechanisms underlying Charcot arthropathy development. However, verification of these mechanisms will require additional studies, integrating SNPs, methylation pattern and transcriptome analyses which could lead to better understanding and preventing Charcot arthropathy.

## Methods

### Study participant’s characteristics

This study was performed with the approval of the Medical University in Gdansk Research Ethics Committee and all the participants gave written informed consent. It was carried out in accordance with the Declaration of Helsinki ethical principles on human studies and all the experiments was conducted with the approved guidelines and regulations. The study group consisted of patients of the Diabetology Clinique at the Medical University of Gdansk. Patients were recruited to the study on the basis of diagnosed Type I or Type II diabetes, in accordance with applicable criteria of the Polish Diabetes Association.

Patients were classified into study groups based on the occurrence of clinical features, characteristic of Charcot arthropathy, neuropathy or diabetes. Qualification for Charcot arthropathy group required the presence of the following symptoms: unilateral redness, edema of the foot, increased foot temperature compared to the other limb and radiological changes. Other diseases that have similar symptoms, like gout, deep vein thrombosis or inflammation of the bone, were excluded. Patients were classified into the neuropathy group based on the sensorimotor neuropathy examination. Patients diagnosed with other potential causes of damage to the peripheral nervous system, such as Vitamin B12 deficiency, use of neurotoxic drugs, hereditary neuropathy, abuse of alcohol or heavy metal poisoning, have been excluded. A group of people with diabetes consisted of patients without neurological changes or other disqualifying conditions.

### SNPs Genotyping

Three ml of blood were collected from the cubital vein into the EDTA containing tubes (BD Vacutainer K_2_EDTA) from each patient. After collecting the blood, the serum was separated from whole blood by centrifugation at 2500 × *g*, for 10 minutes. Genomic DNA was isolated using QIAamp DNA Blood Mini Kit (Qiagen). The DNA concentration was assayed using the NanoDrop™SpectroPhotometr.

Genotyping was performed by Restriction Fragment Length Polymorphism (RFLP) for all alleles, except for *RANKL* 290C/T. Products were electrophoresed in agarose gel in the sodium boric acid buffer^29^. Primers and corresponding amplicons, as well as restriction enzymes used for genotyping, are summarized in Table 2. In general, the PCR mixture contained 20 ng of genomic DNA in 20 μl total volume, prepared accordingly to manufacturer’s instructions (Taq DNA Polymerase, Roche Life Science). Amplification was carried out as suggested by manufacturer with the exception of annealing temperature, which has been optimized for each pair separately (Table 2). For digestion with a restriction enzyme, 10 μl of PCR products were used.

**Table 2:** Summary of primers and amplicons used for SNPs genotyping.

Gene	SNP	Primer sequence	Tm [°C]	PCR length [bp]	RFLP	Reference
OPG	rs3134069 (245T/G)	CTGGAGACATATAACTTGAACA	53	300	HinfI	21
		CCATCATCAAAGGGCTATTGGT				
	rs2073617 (950T/C)	GTTCCTCAGCCCGGTGGCTTTT	65	342	HincII	
		TGTGGTCCCCGGAAACCTCAGG				
	rs2073618 (1181G/C)	ACTTCCTGTTGCCGGGACGCT A	60	147	SmoI	
		TACCACGAGCGCGCAGCACCTCA				
	rs3102734 (1217C/T)	GCAGGCGATACTTCCTGTT	59	298	BsuRI	
		GTTTCCTGCTCCAGCCTAAC				
	rs7844539 (6890A/C)	GTATTGAATAGACTCTCAGAAA	51	381	BclI	
		AACTAAACATACATGCAGTCTT				
RANKL	rs9533155 (693G/C)	TGGTCAGCAACTTCCTTCTG	60	551	BseDI	30
		GACATTCCTCCTGCATCCAT				
	rs9533156 (643C/T)	TGGTCAGCAACTTCCTTCTG	60	551	TspRI	
		GACATTCCTCCTGCATCCAT				
	rs9525641 (290C/T)	CCAAACTAGAATGGATGCAGGAGG	59	149	Minisequencing	This study
		TACTCCAGTGGTTCCAGACTCCCC				
RANK	rs1805034 (421C/T)	GGCCACTGACCTGTCTCTTG	59	99	Csp6I	31
		GACAGACGCACACATCCAAC		77		
				25		
	rs35211496 (575C/T)	CCAAAGCACTGAACCACCTT	58	107	SsiI	
		TGGCAGAGAAGAACTGCAAA				

### Minisequencing

The PCR amplicon for *RANKL* 290C/T was generated with primers described in Table 2. The reaction was purified with Exo-SAP kit and submitted to sequencing. The reaction was performed using 5′-TTT TTT TTT T CAAA GGT GTC CTC TGC GTC TTC-3′ primer and the ABI PRISM® SNaPshot® Multiplex Kit [Applied Biosystems]. Products were subjected to capillary electrophoresis run in the ABI Prism 310 [Applied Biosystems] by using Hi-Di™ Formamid, *Gene Scan™120 LIZ™ Size Standard*. Electrophoresis was carried out in POP-4 polymer under denaturing conditions of 60 °C for 20 minutes. Chromatograms were analysed using the Gene Scan™v.3.1.2. software.

### Biochemical analysis of OPG and sRANKL in the blood serum

OPG and sRANKL (soluble RANKL) levels in the blood serum were measured using enzyme-linked immunosorbent assays. Commercial ELISA kits for Human Osteoprotegerin (BioVendor RD194003200) and Human sRANKL (ampli-sRANKL kits Biomedica BI-20452) were used according to the manufacturer’s protocol. The detection limit was 0.1 pmol/l and 0.02 pmol/l for OPG and sRANKL, respectively.

### Statistical analysis

Calculations and statistical analysis of polymorphisms occurrence and difference in occurrence was performed with packages genetics^32^ and LDheatmap^33^. Significance of differences in protein levels measured by ELISA was verified by an unpaired Student *t* test (unpaired). Hierarchical clustering has been performed in R using heatmap.2 function in the package ggplots^34^.

## References

[1] United Nations, A/RES/61/225: World Diabetes Day, 83rd Plenary Meeting, 20th December 2006 (2006).

[2] International Diabetes Federation Diabetes atlas 7th edition. Available from: www.idf.org/diabetesatlas (2015).

[3] World Health Organization 2015. Diabetes. Fact sheet N°312Updated January 2015. Geneva, Switzerland: World Health Organization (2015).

[4] Vinik AI (1999). Diabetic neuropathy: pathogenesis and therapy. Am J Med..

[5] Tesfaye S, Selvarajah D (2012). Advances in the epidemiology, pathogenesis and management of diabetic peripheral neuropathy. Diabetes Metab Res Rev..

[6] Boulton AJM, Gries FA, Jervell JA (1998). Guidelines for the diagnosis and outpatient management of diabetic peripheral neuropathy. Diabet Med..

[7] Jeffcoate WJ, Harding KG (2003). Diabetic foot ulcers. Lancet..

[8] Bruhn-Olszewska B (2012). Molecular factors involved in the development of diabetic foot syndrome. Acta Biochim Pol..

[9] Ndip A, Wilkinson FL, Jude EB, Boulton AJ, Alexander MY (2014). RANKL–OPG and RAGE modulation in vascular calcification and diabetes: novel targets for therapy. Diabetologia..

[10] Kearns AE, Khosla S, Kostenuik PJ (2008). Receptor activator of nuclear factor κB ligand and osteoprotegerin regulation of bone remodeling in health and disease. Endocr Rev..

[11] Khosla S (2001). Minireview: The opg/rankl/rank system. Endocrinology..

[12] Boyle WJ, Simonet WS, Lacey DL (2003). Osteoclast differentiation and activation. Nature..

[13] Mountzios G (2007). Abnormal bone remodeling process is due to an imbalance in the receptor activator of nuclear factor-κB ligand (RANKL)/osteoprotegerin (OPG) axis in patients with solid tumors metastatic to the skeleton. Acta Oncol..

[14] Kiechl S (2006). The osteoprotegerin/RANK/RANKL system: a bone key to vascular disease. Expert Rev Cardiovasc Ther..

[15] Petrova NL, Shanahan CM (2014). Neuropathy and the vascular-bone axis in diabetes: lessons from Charcot osteoarthropathy. Osteoporos Int..

[16] Jeffcoate WJ (2005). Theories concerning the pathogenesis of the acute Charcot foot suggest future therapy. Curr Diab Rep..

[17] Pitocco D (2009). Association between Osteoprotegerin G1181C and T245G Polymorphisms and Diabetic Charcot Neuroarthropathy A case-control study. Diabetes Care..

[18] Korzon‐Burakowska A (2012). Osteoprotegerin gene polymorphism in diabetic Charcot neuroarthropathy. Diabet Med..

[19] Rogers LC (2011). The Charcot foot in diabetes. Diabetes Care..

[20] Wuyts W (2001). Evaluation of the role of RANK and OPG genes in Paget’s disease of bone. Bone..

[21] Langdahl BL, Carstens M, Stenkjaer L, Eriksen EF (2002). Polymorphisms in the osteoprotegerin gene are associated with osteoporotic fractures. J Bone Miner Res..

[22] Petrova NL, Petrov PK, Edmonds ME, Shanahan CM (2015). Inhibition of TNF-α reverses the pathological resorption pit profile of osteoclasts from patients with acute Charcot osteoarthropathy. J Diabetes Res..

[23] Ndip A (2011). The RANKL/RANK/OPG signaling pathway mediates medial arterial calcification in diabetic Charcot neuroarthropathy. Diabetes..

[24] Chen C (2014). MiR‐503 Regulates Osteoclastogenesis via Targeting RANK. J Bone Miner Res..

[25] Murakami Y (2006). Comprehensive analysis of microRNA expression patterns in hepatocellular carcinoma and non-tumorous tissues. Oncogene..

[26] Reddy MA, Zhang E, Natarajan R (2015). Epigenetic mechanisms in diabetic complications and metabolic memory. Diabetologia..

[27] Park LK (2014). Genome-wide DNA methylation analysis identifies a metabolic memory profile in patient-derived diabetic foot ulcer fibroblasts. Epigenetics..

[28] Esteller M (2011). Non-coding RNAs in human disease. Nat Rev Genet..

[29] Brody JR, Kern SE (2004). Sodium boric acid: a Tris-free, cooler conductive medium for DNA electrophoresis. Biotechniques..

[30] Mencej S, Preželj J, Kocijančič A, Ostanek B, Marc J (2006). Association of TNFSF11 gene promoter polymorphisms with bone mineral density in postmenopausal women. Maturitas..

[31] Choi JY (2005). Genetic polymorphisms of OPG, RANK, and ESR1 and bone mineral density in Korean postmenopausal women. Calcif Tissue Int..

[32] Warnes, G., Gorjanc, G., Leisch, F. & Man, M. genetics: Population Genetics. R package version 1.3.8.1. https://CRAN.R-project.org/package=genetics.

[33] Shin, J-H., Blay, S., McNeney, B. & Graham, J. LDheatmap: An R Function for Graphical Display of Pairwise Linkage Disequilibria Between Single Nucleotide Polymorphisms (2006).

[34] Warnes, G. R. *et al*. gplots: Various R Programming Tools for Plotting Data. R package version 2.17.0. https://CRAN.R-project.org/package=gplots (2015).

